# *In-vivo *validation of a new non-invasive continuous ventricular stroke volume monitoring system in an animal model

**DOI:** 10.1186/cc10306

**Published:** 2011-07-11

**Authors:** Maurits K Konings, Paul F Grundeman, Henk G Goovaerts, Maarten R Roosendaal, Imo E Hoefer, Pieter A Doevendans, Frank E Rademakers, Wolfgang F Buhre

**Affiliations:** 1 Department of Medical Technology and Clinical Physics, University Medical Center Utrecht, 100 Heidelberglaan, Utrecht 3584 CX, The Netherlands; 2 Department of Experimental Cardiology, University Medical Center Utrecht, 100 Heidelberglaan, Utrecht 3584 CX, The Netherlands; 3 Goovaerts Instruments, 15 Bruinjoost, Kortenhoef 1241 HN, The Netherlands; 4 Department. of Cardiology, University Hospitals Leuven, 49 Herestraat, Leuven 3000, Belgium; 5 Department of Anesthesiology, University Medical Center Utrecht, 100 Heidelberglaan, Utrecht 3584 CX, The Netherlands

## Abstract

**Introduction:**

Recently, a non-invasive, continuous ventricular stroke volume monitoring system using skin electrodes has been developed. In contrast to impedance-based methods, the new technique (ventricular field recognition) enables measurement of changes in ventricular volume. A prototype using this new method was built (the hemologic cardiac profiler, HCP) and validated against a reference method in a pig model during variations in cardiac output.

**Methods:**

In six Dalland pigs, cardiac output was simultaneously measured with the HCP (CO-HCP), and an invasive ultrasonic flow-probe around the ascending aorta (CO-FP). Variations in CO were achieved by change in ventricular loading conditions, cardiac pacing, and dobutamine administration. Data were analysed according to Bland-Altman analysis and Pearson's correlation.

**Results:**

Pearson's correlation between the CO-HCP and the CO-FP was r = 0.978. Bland-Altman analysis showed a bias of - 0.114 L/minute, and a variability of the bias (2 standard deviations, 2SD) of 0.55 L/minute.

**Conclusions:**

The results of the present study demonstrate that CO-HCP is comparable to CO-FP in an animal model of cardiac output measurements during a wide variation of CO. Therefore, the HCP has the potential to become a clinical applicable cardiac output monitor.

## Introduction

Measurement of cardiac output (CO) is important in the hemodynamic management of peri-operative and critically ill patients. Pulmonary artery thermodilution CO monitoring using the pulmonary artery catheter is still the clinical reference technique of CO-monitoring, but has major disadvantages, mainly associated with the high invasiveness of the technique [1]. There is a need for a non-invasive, precise, continuous, and operator-independent technique of CO measurement. During the last years a number of minor-invasive or even non-invasive techniques have been developed. Non-invasive ultrasound Doppler techniques like transthoracic echocardiography have been developed, but require skilled operators, and, therefore, are not suited for monitoring CO continuously. In contrast, operator independent techniques like oesophageal Doppler are not tolerated well in the awake patient, which is an important limitation in the intensive care setting. Bioimpedance techniques have been evaluated, with results ranging from acceptable to inconclusive [2]. Furthermore, bioreactance techniques have been tested in a limited number of clinical situations.

We recently developed a new technique for non-invasive continuous cardiac output monitoring, which we will refer to as 'Ventricular Field Recognition', based on measurement of spatial patterns of voltage changes distributed over the thoracic skin [3]. Basically, the Ventricular Field Recognition method is based on the finding that, if a weak electric current is applied over the thorax, emptying and filling of the ventricles during the cardiac cycle give rise to two-dimensional spatial patterns of voltage changes on the thoracic skin, that are distinctly different from the patterns due to filling and emptying of the *atria*. This is caused by the fact that the ventricles are situated at other locations inside the thorax than the atria, and hence, the location of the ventricular "epicenter" of the changes in the applied current field, due to ventricular volume changes, differs from the location of the atrial "epicenter" corresponding to atrial volume changes. The changes in the applied current field, associated with ventricular emptying, represent a particular two-dimensional spatial pattern of voltage changes on the thoracic skin, which we refer to as the ventricular "fingerprint". This ventricular fingerprint can be recognized, and distinguished from the atrial fingerprint, provided that a sufficient number of measuring electrodes are distributed over the thoracic surface at the optimal places.

Although our Ventricular Field Recognition method depends on the differences in specific conductivity between blood and other tissues, it is not based on Impedance cardiography (ICG), but instead uses eight independent and simultaneous voltage measurements to probe the spatial distribution of voltages over the thoracic skin. Furthermore, unlike ICG or bioreactance-based techniques [4], we retrieve the volume changes (as a function of time during the cardiac cycle) of exclusively the ventricles, instead of volume changes of the entire heart [5]. In the current study we perfomed validation experiments in Dalland pigs against the gold standard, an ultrasonic flow-probe around the ascending aorta, under defined changes in loading conditions, cardiac pacing and inotropic stimulation by dobutamine.

## Materials and methods

### Animals

All experiments were performed in accordance with the "Guide for the Care and Use of Laboratory Animals" prepared by the Institute of Laboratory Animal Resources and with prior approval by the Animal Experimentation Committee of the Faculty of Medicine, Utrecht University, the Netherlands. The study was performed on six Dalland Landrace pigs (IDDLO, Lelystad, The Netherlands), in a weight range from 39 to 82 kg.

### Anesthesia

Three days before the start of the experiment a daily dose of 75 mg clopidogrel and a bolus of 500 mg acetylsalicylic acid was administered. After overnight fasting, anaesthesia was induced using Ketamin (10 mg/kg), midazolam (0.5 mg/kg), and atropin (0.04 mg/kg) and additional thiopentone (5 mg/kg) immediately before intubation. Muscle relaxation was achieved by bolus injection of 0.1 mg kg^-1 ^pancuronium bromide. After intubation, controlled positive pressure ventilation was installed with (tidal volume 10 ml/kg, 12 breaths/minute) an inspiratory oxygen concentration of 0.5. A venous catheter was placed in an ear vein for continuous administration of saline and anesthetic drugs. Anesthesia was maintained by continuous infusion of midazolam (1.0 mg · kg^-1^ · h^-1^), and sufentanil citrate (2 μg · kg^-1^ · h^-1^). In addition, pancuronium bromide was administered continuously (0.1 mg · kg^-1^ · h^-1^). Before surgery, four times (with an interval of 15 minutes), a bolus of 0.05 mg/kg propronolol was given.

### Multi-electrode method to separate ventricular from atrial filling

A set of nine electrodes was attached to the thorax, according to a fixed scheme of electrode positions. The relative positions of the nine electrodes have been optimized in such a way that the ventricular volume-time curve can be retrieved [3]. With the use of ultrasound (Philips i33 Cardiograph, Philips Medical Systems, Eindhoven, The Netherlands) the contour of the heart was estimated and drawn on the thoracic skin using a permanent marker. The "gauge point" (GP) on the skin was defined as the position of the mitral valve projected on the skin, estimated on the basis of the contour of the heart on the thorax. Subsequently, each of the nine measuring electrodes was placed on the thorax at a specific relative position with respect to the point GP, as specified in Table 1.

**Table 1:** Electrode positions, rendered as coordinates (X, Y) on the thoracic skin of the animal

Electrode #	Position X (in mm) Horizontal position (left-right) **with respect to Gauge Point (GP)**. Orientation: The left-lateral side of the thorax has positive X values	Position Y (in mm) Vertical position (cranial-caudal) **with respect to Gauge Point (GP)**. Orientation: The cranial end of the thorax has positive Y values
1	24.5	9.1
2	-28.1	34.2
3	-36.0	-16.2
4	64.9	28.8
5	-59.5	46.8
6	-27.0	-81.1
7	-41.4	158.7
8	-9.0	-131.6
9	-86.5	61.3

The 2D positions of the electrodes are defined as relative distances with respect to the Gauge Point (GP). These relative distances are rendered as displacements in the horizontal direction (X, left-right) and the vertical direction (Y, cranial-caudal), respectively, as measured along the (curved) thoracic skin. These nine electrodes can be applied conveniently using a single compound electrode sticker, in which all electrodes are at their correct relative positions with respect to each other.

### Measuring equipment

The nine measuring electrodes mentioned above were connected to the Hemologic Cardiac Profiler system, which contains eight current source/demodulator units based as described earlier in more detail [6,7], and a set of two personal computers (PCs). One PC serves for data acquisition and storage, and the second for data processing and control. The current sources produce an alternating current of 8 mApp at a fixed frequency in the range between 55 kHz and 91 kHz. The current injection can be selected to take place at both forelegs and hind legs simultaneously or separately. The resulting voltage distribution on the thorax is measured by a set of measuring electrodes, connected to the eight demodulator systems mentioned above, which produce eight independent input signals for the data-acquisition computer. As a reference method to measure cardiac output, a bi-directional ultrasonic flow probe was attached around the ascending aorta, using a Transonic T206 small animal flowmeter (Transonic Systems Inc, Ithaca, New York, USA). The flowmeter output was logged on to a notebook PC to enable accurate simultaneous comparison between calculated data from the HCP and data obtained from the flowmeter. The HCP produces one CO measurement every 20 seconds.

### Manipulation of cardiac output

During each session, the following steps were taken to induce changes in cardiac output:

- Decrease in venous return induced by a vena cava obstruction. For this purpose, a 3-0 prolene wire (Ethicon Inc., Somerville, New Jersey, USA) was placed around the vena cava. A step-wise decrease of cardiac output, as monitored by the ultrasonic flow-probe, could be obtained by step-wise tightening of the prolene wire.

- Increase in heart rate by external cardiac pacing (Q-stim, Biosense Webster Inc., Diamond Bar, California, USA) connected to the stimulator output of a Physio-control Lifepack 9 defibrillator (Physio-control Inc., Redmond, Washington, USA.)

- Inotropic stimulation by administration of dobutamine (5 μg/kg/minute b.w. i.v.)

### Data acquisition and averaging

Each data point in the results section represents a single period of 20 seconds. The HCP produces one single CO value per 20 seconds, whereas the ultrasonic flow probe (FP) produces beat-by-beat values. Therefore, the data from the FP were averaged to produce a single CO value per 20 seconds. Only data points that represented a stable situation (that is, a constant CO value during 20 seconds) were incorporated into the data sets in the Results section. During the vena cava obstruction experiments, after each increase of the tightening of the prolene wire, the experimenter waited until a stable CO value was reached, and then recorded a single data point of 20 seconds. For each of the six animals, seven different levels of prolene wire tightening were used, resulting in 42 data points from the vena cava obstruction experiments.

During the external cardiac pacing experiments, three different heart rates were produced: 100 bpm, 120 bpm, and 140 bpm. For each of these three heart rates, one data point of 20 seconds was produced. For one specific animal, one single data point was excluded because this animal failed to endure cardiac pacing of 140 bpm. As a result, the cardiac pacing experiments produced 17 data points for six animals. During the inotropic stimulation experiments with dobutamine, for each animal one data point was recorded at each of the following three moments in time: at one minute, at four minutes, and at seven minutes after the start of the continuous dobutamine administration. One specific animal, however, failed to endure prolonged dobutamine administration and, subsequently, the dobutamine administration was stopped after five minutes, and, hence, no data point corresponding to seven minutes of dobutamine administration was recorded for this specific animal. Therefore, 17 data points were recorded for the dobutamine experiments for six animals.

For the total study, this amounts to 76 independent data points. In both instances in which there was an inability to obtain a data point, this was only due to the condition of the animal.

### Initial calibration

For each animal, an initial, once-only calibration of the Hemologic Cardiac Profiler (HCP) was performed on the basis of the readings of the invasive bi-directional flow probe. The flow probe was calibrated on a regular basis using an *in-vitro *set-up.

### Statistical analysis

The study design was developed under the guidance of the head of the Department of Statistics (Ingeborg van de Tweel) for the Life Sciences of Utrecht University, and approved by the Animal Experimentation Committee of the Faculty of Medicine, Utrecht University, The Netherlands. Pearson's correlation was used to evaluate the correlation between CO measurements from the HCP and CO measurements from the ultrasonic flow-probe around the ascending aorta. The fact that multiple measurement values exist per animal, was taken into account by basing the correlation coefficient and the 95% confidence intervals on only the data points corresponding to the maximum intensity stage of each type of manipulation (that is, maximal tightening of the prolene wire during vena cava constriction, maximum heart rate (140 bpm) during cardiac pacing, and the longest period (seven minutes) of continuous dobutamine administration), for each of the six animals. Furthermore, an additional regression plot was added, in which all CO values was rendered as a percentage of the initial baseline CO of each animal. As a result, the regression line passes through (100,100). This enables rendering the 95% confidence interval (95% CI) lines into the plot.

Furthermore, Bland-Altman analysis was used. Three ranges of CO values were defined: (i) 0 to 2 L/minute, (ii) 2 to 4 L/minute, and (iii) over 4 L/minute. For each of these three ranges, bias and variability of the bias (2 standard deviations, 2SD), and lower and upper limits of agreement, have been calculated. In these calculations, the presence of multiple measurements per animal was taken into account by using the Components of Variance technique [8].

Furthermore, the performance of the new HCP system was evaluated using four quality criteria proposed by Squara *et al*. [9]: (i) bias, (ii) random error of measurements of each modality of measurement (HCP and Flow Probe), estimated by the variability (2SD) during a steady-state situation at baseline level before the start of the manipulations of the CO, (iii) response time, and (iv) reliability for detecting directional changes.

For all changes larger than the overall SD, the directional changes were defined using the categories "concordant" (changes in HCP and FP both larger than SD and both pointing in the same direction), "discordant" (changes in HCP and FP both larger than SD but pointing in different directions), false positive (change in HCP larger than SD, but change in FP smaller than SD), and false negative (change in HCP smaller than SD, but change in FP larger than SD).

## Results

Experiments were performed in six animals in the weight range from 33 kg to 82 kg. An overview of the six animals is given in Table 2.

**Table 2:** Animal overview

Animal #	Type	Gender	Weight (kg)	Age (weeks)
1	Dalland Pig	Female	45,.	12
2	Dalland Pig	Female	45	12
3	Dalland Pig	Female	44	12
4	Dalland Pig	Female	42.6	12
5	Dalland Pig	Female	33	10
6	Dalland Pig	Female	82	19

### Measured cardiac output data

In total, 76 pairs of cardiac output measurements were performed.

**Figure 1:**
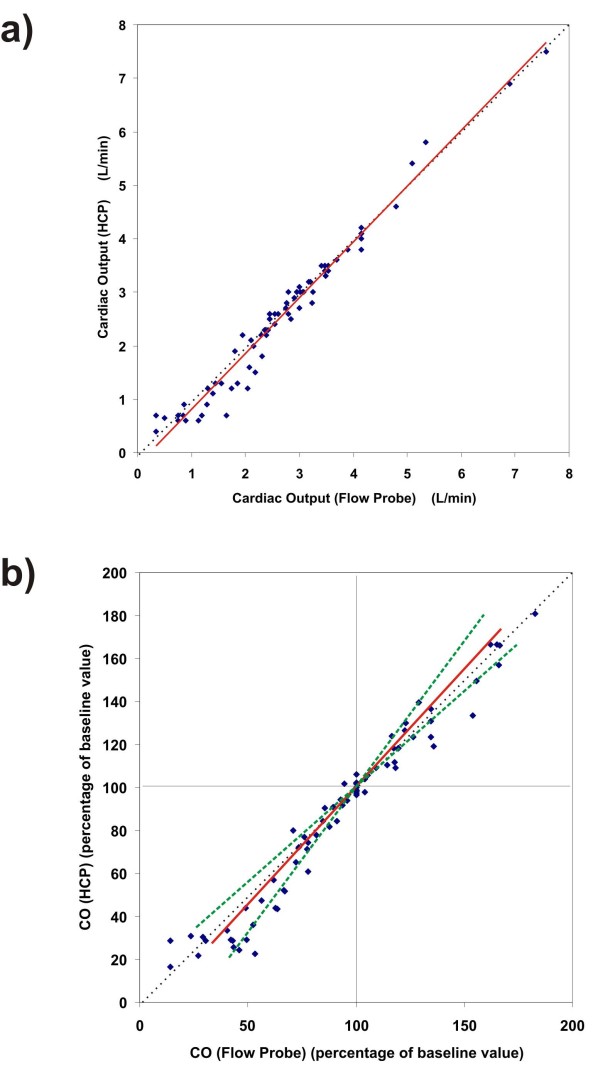
**Collective results of the experiments on all six animals**. (**a**) Here, each CO value, produced by the new HCP system at some moment in time during the experiments, is plotted as function of the corresponding CO value as measured by the invasive flow probe at the same moment in time. A trend line (linear regression, solid red line) has been added; slope = 1.045 (95% CI is 0.96 to 1.14). Pearson's correlation was r = 0.978. An identity line (dotted thin line) has been added. (**b**) Same as a), but now all CO values are rendered as a percentage of the initial baseline CO value of each animal. This enables rendering of the 95% CI boundary lines (dashed green lines). Here, r = 0.972 and slope = 1.062 (95% CI is 0.94 to 1.18).

The coefficient of correlation between CO-HCP and CO-FP was r = 0.978 (see Figure 1), and the slope was 1.045 (with 95% confidence interval from 0.96 to 1.14). The coefficient of correlation and the 95% confidence interval have been corrected for the multiple data points per animal.

**Figure 2:**
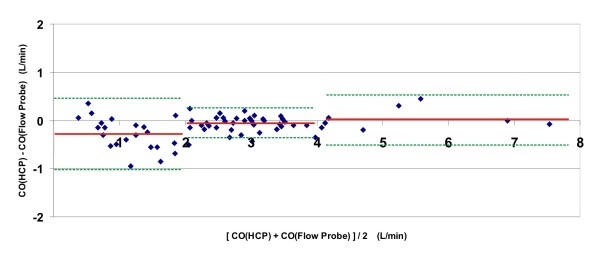
**Bland-Altman plot of the collective results of all six animals**. For each moment in time during the experiments that a cardiac output value was produced by the HCP system, the difference between the CO, according to the HCP, and the CO, according to the flow probe, was plotted as a function of the average of both CO's at that moment. For each of three intervals along the x-axis (region 1: CO < 2 L/minute; region 2: CO between 2 and 4 L/minute; region 3: CO > 4 L/minute), the bias is rendered (red solid line), along with the limits of agreement (green dotted lines).

Bland-Altman analysis (Figure 2) showed a bias of - 0.28 L/minute and a variability of the bias (2SD) of 0.74 L/minute in the range of 0 to 2 L/minute, a bias of - 0.05 L/minute and a variability of the bias (2SD) of 0.31 L/minute in the range of 2 to 4 L/minute, and a bias of 0.02 L/minute and a variability of the bias (2SD) of 0.54 L/minute in the range above 4 L/minute. Lower limits of agreement were -1.02 L/minute in the range of 0 to 2 L/minute, - 0.36 L/minute in the range of 2 to 4 L/minute, and - 0.53 L/minute in the range above 4 L/minute. Upper limits of agreement were 0.47 L/minute in the range of 0 to 2 L/minute, 0.26 L/minute in the range of 2 to 4 L/minute, and 0.56 L/minute in the range above 4 L/minute.

For all data combined, mean bias was - 0.114 L/minute (or -3.6%), variability of the bias (2SD) was 0.55 L/minute (or 17.6%), lower limit of agreement was - 0.67 L/minute, and upper limit of agreement was 0.44 L/minute.

**Figure 3:**
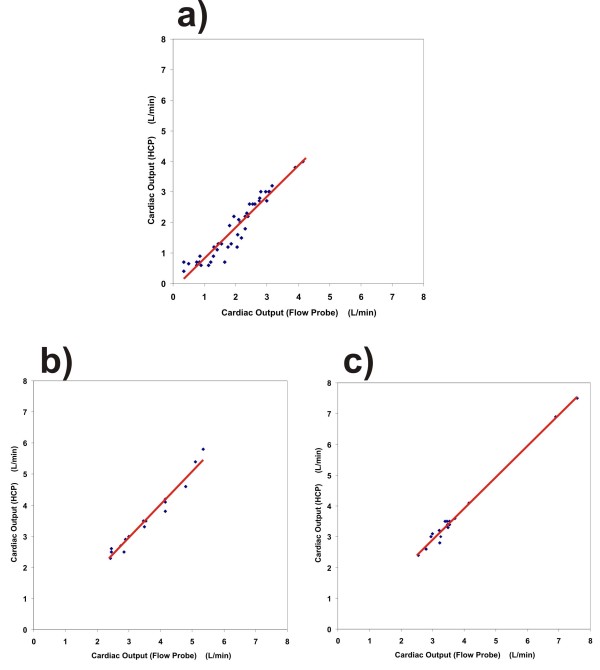
**Regression plots for three methods of manipulating the cardiac output**. **a**) Flow restriction in the vena cava inferior: r = 0.955, slope = 1.02, and 95% CI is 0.92 to 1.12. **b**) Cardiac pacing: r = 0.982, slope = 1.06, and 95% CI is 0.95 to 1.17. **c**) Dobutamine administration: r = 0.981, slope = 1.01, and 95% CI is 0.95 to 1.07. For each of the three plots, a regression line (red solid line) has been added.

For each of the manipulations, a separate plot was produced with corresponding confidence intervals (95%CI), see Figure 3.

Hemodynamic data during baseline conditions and the maximum intensity stages of each manipulation are rendered in Table 3.

**Table 3:** Hemodynamic data for six animals

	Baseline	Vena cava constriction (maximal)	Pacing (140 bpm)	Dobutamine (7 minutes)
HR (bpm)	54 ± 8	63 ± 11	140 ± 1	89 ± 14
MAP (mmHg)	118 ± 17	96 ± 27	113 ± 23	138 ± 26

HR(bpm), heart rate (beats per minute); MAP(mmHg), mean arterial pressure (millimeter of mercury). Data presented as mean ± standard deviation. Data measured at baseline and at the maximum intensity of each of the three CO manipulations.

Furthermore, the performance of the HCP system was evaluated using four quality criteria proposed by Squara *et al*. [9]: (i) *Bias*: overall bias was small (- 0.114 L/minute, or -3.6%). (ii) *Random error of measurements*: during a steady-state situation at baseline level before the start of the manipulations of the CO, the 2SD (precision) within the HCP data was assessed, as well as the 2SD within the FP data during the same period of time. This resulted in 2SD = 0.33 L/minute for the HCP, and 2SD = 0.06 L/minute for the FP. Since the random error of measurement of the FP is very small, and the CO values produced by the FP are very close to the truth, application of the errorgram method described by Critchley and Critchley to account for the errors caused by the reference technique yields virtually the same answers as the 2SD of the HCP alone. (iii) *Response time *is 27 seconds for the HCP: 20 seconds of data acquisition followed by 7 seconds of automated data processing leading to one single CO value. (iv) *Reliability for detecting directional changes*: for all three different manipulations, all CO changes exceeding 0.27 L/minute (the overall SD of the bias) have been analysed; see Table 4.

**Table 4:** Ability of the HCP to track changes in cardiac output correctly, for each of the three methods of CO manipulation

Type of manipulation	Number of changes > SD	Concordant*	Discordant*	False positive*	False negative*
Vena cava constriction	46	42 *(91.3%)*	1 *(2.2%)*	2 *(4.3%)*	1 *(2.2%)*
Cardiac pacing	4	3 *(75.0%)*	0	1 *(25.0%)*	0
Dobutamine	8	7 *(87.5%)*	0	1 *(12.5%)*	0
					
All manipulations combined	58	52 *(89.6%)*	1 *(1.7%)*	4 *(6.9%)*	1 *(1.7%)*

Only changes larger than the overall standard deviation (SD, 0.27 L/minute) have been considered, see subsection 2.8

(*): Data rendered as: number of points (percentage of total number of changes (> SD) in manipulation)

**Figure 4:**
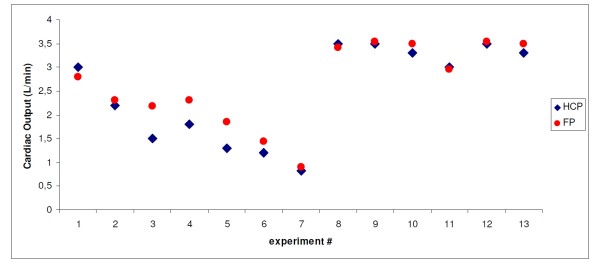
**Example of a series of data points from one single animal**. In this graph, the results of the experiments on animal #3 are rendered, showing the cardiac output as measured by the HCP (CO-HCP, dark blue diamonds), and as measured by the ultrasonic flow probe (CO-FP, red dots). The enumeration of the experiments along the x-axis corresponds to the chronological order in which the measurements have taken place on this animal. Thirteen independent data points have been collected. Data points #1 to #7 represent increasing levels of vena cava constriction; data points #8 to #10 represent three levels of external cardiac pacing (at 100 bpm, 120 bpm, and 140 bpm, respectively), and data points #11 to #13 represent the effects of one minute, four minutes, and seven minutes of continuous dobutamine administration, respectively.

As an example, a series of data points from one single animal (animal #3) has been rendered in Figure 4.

## Discussion

The results of this validation study demonstrate that cardiac output measures obtained with the Hemologic Cardiac Profiler correlate well with measures obtained with direct aortic blood flow measurements (the accepted gold standard), after an initial calibration with the reference method.

### Comparison between HCP and ICG

A principal difference between the HCP and ICG or bioreactance-based techniques, is that the HCP performs a direct measurement of ventricular volume changes, instead of total heart volume changes. Therefore, the HCP, theoretically, provides a better basis for assessment of stroke volume, because total heart volume variation is not necessarily a reliable indicator of ventricular stroke volume [5]. Furthermore, in comparison to ICG or bioreactance-based techniques, the HCP may have, at least theoretically, the following advantage:

The voltage signal measured in every ICG system is caused by the combined effects of volume changes in different intrathoracic compartments during the cardiac cycle, such as the intracardiac cavities, aorta, superior and inferior vena cava, and pulmonary circulation. From all these factors, ICG attempts to distill the stroke volume using a single input voltage stream. From a mathematical point of view, however, this single voltage input stream is insufficient to balance the many unknown variables inside the thorax. Some ICG-like techniques (such as, for example, Woltjer *et al*. [10]) use multiple electrodes, but - in contrast to our method - still measure only one single voltage, because the leads of many of these electrodes are interconnected to form a single input channel. In contrast, with the new technique (HCP), nine electrodes produce eight independent and simultaneous voltage input streams, connected to eight independent voltage demodulator units.

In this study, an initial calibration process using the flow probe was performed for each animal, because of the unknown attenuation of signals between the heart and the skin due to inter-individual differences in thoracic wall thickness, (such as differences in thickness of sub-cutaneous fat and muscle layers). In the future, however, the HCP should be able to perform an auto-calibration as a stand-alone technique [3]. In this auto-calibration procedure, the measuring electrodes will be used temporarily for current injection. In an *in vitro *study and in computer models, we have shown that, invoking the reciprocity theorem of electromagnetic fields, this procedure is capable of assessing the attenuation of signals between the heart and the skin. Therefore, in the future, this auto-calibration could potentially take away the need for an initial calibration using ultrasound. Furthermore, recently a small modification of the HCP has been developed that no longer needs the identification of a "gauge point" (GP) on the thorax and, hence, eliminates the need for ultrasound to find the heart contour. In future clinical studies, the autocalibration process and the GP auto- determination must be validated.

We used direct aortic blood flow measurement of cardiac output (CO-FP) as the gold standard. In contrast to thermodilution cardiac output, direct aortic blood flow measurement (CO-FP) has no relevant mean error, thus the measured difference in cardiac output represents the real difference. For all data combined, mean bias was - 0.114 L/minute (or -3.6%), and variability of the bias (2SD) was 0.55 L/minute (or 17.6%.) Thus we can conclude that the mean error between a true reference technique (ultrasonic flow probe around the aorta) and the new technique demonstrates satisfactory validity.

Furthermore, we induced a number of variations in cardiac output, which are either based on changes in cardiac preload (venous inflow), or an increase in heart rate and inotropy (ß-stimulation), to investigate the agreement over a wide variation of CO-values. These variations in cardiac output are common in the clinical setting and the measurement technique should be able to track them. Our data suggest that the validity of the new technique is independent from the absolute cardiac output value above absolute cardiac output values of more than 2 L/minute. We observed a systematic underestimation (-0.28 L/minute, *P *< 0.05) of cardiac output by the HCP in the range below 2 L/minute, as can be seen from the regression line in Figure 1. Further analysis of the performance of the HCP revealed that this underestimation in the range below 2 L/minute was caused by sub-optimal positions of the thoracic electrodes with respect to each other. In clinical practice, CO values below 2 L/minute are not expected to happen in the adult patient population.

## Conclusions

In an animal model we demonstrated that the Hemologic Cardiac Profiler was able to track changes in cardiac output. Therefore, we see a potential for this new technology to be useful in the clinical setting.

## Key messages

• In an animal model we demonstrated that the new technique of cardiac output monitoring (HCP) is able to track changes in cardiac output.

• In contrast to impedance-based methods, the new system (HCP) enables specific measurement of changes in ventricular volume, instead of changes in total heart volume.

• The HCP produces eight independent and simultaneous voltage input streams, connected to eight independent voltage demodulator units. This enables recognition of the effect of ventricular volume changes in the applied field on the thoracic skin.

• An Ultrasonic Flow Probe (FP) around the aorta was used as the gold standard.

• In a study on six pigs, Pearson's correlation between the CO-HCP and the CO-FP was r = 0.978. Bland-Altman analysis showed a bias of - 0.114 L/minute, and variability of the bias (2SD) of 0.55 L/minute.

## Abbreviations

ANOVA: analysis of variance; CI: confidence interval; CO: cardiac output; CO-FP: cardiac output according to the ultrasonic flow probe; CO-HCP: cardiac output according to the HCP; FP: Ultrasonic Flow Probe; GP: gauge point; HCP: Hemologic Cardiac Profiler; ICG: Impedance Cardiography; MAP: mean arterial pressure.

## Competing interests

The authors declare that they have no competing interests.

HGG has received some reimbursements for advice concerning the technical setup of the experiments, and HGG has some financial interest in an organization that potentially may profit from publication of this manuscript in the future.

## Authors' contributions

MKK drafted the paper and performed experiments, data acquisition and analysis. PFG designed the experimental set-up and performed experiments. HGG designed the electronic instrumentation and assisted during experiments. MRR and IEH contributed to the experimental set-up. PAD and FER contributed to the design of the study and the paper. WFB contributed to the manuscript and the interpretation and statistical analysis of data and the discussion. All authors read and approved the final manuscript.
